# Comprehensive studies of temperature and frequency dependent dielectric and a.c. conducting parameters in third generation multi-component glasses

**DOI:** 10.1039/c8ra04214d

**Published:** 2018-07-16

**Authors:** Namrata Chandel, M. M. A. Imran, Neeraj Mehta

**Affiliations:** Glass Science Laboratory, Department of Physics, Banaras Hindu University Varanasi-221005 India dr_neeraj_mehta@yahoo.co.in; Department of Physics, Sunbeam College for Women Varanasi-221005 India; Department of Physics, Faculty of Science, Al-Balqa Applied University Al-Salt 19117 Jordan

## Abstract

The dielectric relaxation and thermally assisted a.c. conduction play an important role in understanding the conduction mechanism in chalcogenide glasses. These two phenomena are often the deciding factors of the suitability of chalcogenide materials for particular device applications. Dielectric relaxation studies are important to understand the nature and origin of dielectric losses, which, in effect, may be useful in the determination of structure and defects in solids. The study of thermally assisted a.c. conduction can be used as a tool to understand the nature of defect states and the estimation of their density of defect states. In this paper, therefore, we have studied the metal-induced effects of cadmium (Cd), indium (In) and antimony (Sb) on dielectric relaxation and thermally activated a.c. conduction in ternary Se_80_Te_18_Sn_2_ glass. The density of charged defect states in quaternary Se_80_Te_8_Sn_2_M_10_ alloys is found to vary with the electro-negativity difference (*ξ*_M_ − *ξ*_Te_) of the foreign element M and Te. Further analysis shows that the increasing sequence of the density of charged defects is explained in terms of variation in the lone-pair electrons after the incorporation of Cd, In and Sb.

## Abstract 2

The value of *N* increases with the rise in LP as a signature of metal-induced effects of Cd, In and Sb.

## Graphical abstract

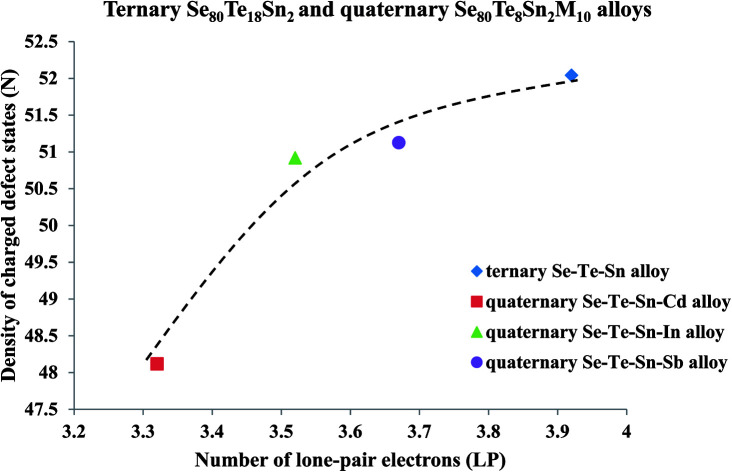

## 1. Introduction

Glasses are distinct from ordinary non-crystalline materials. Unlike usual non-crystalline materials, they possess a glass transition.^1^ In the family of glasses, the semiconducting chalcogenide crystals and glasses have drawn considerable interest in areas of solid-state physics, chemistry and materials science because of their exciting commercial applications and properties.^2–6^ Recently, chalcogen rich non-oxide glasses have found use in potential applications in phase-change data storage,^2^ electronic tongues,^3^ and memory cells.^4^

These materials have recently come under scientific scrutiny due to the strong relation between their electronic properties and disordered structure.^7^ A relevant and complete understanding of their electrical transport properties is, therefore, an area of on-going research activity.^8,9^ The basic goal behind such studies is to explore the electrical properties of chalcogenide glasses for further practical utilization. A common characteristic of semiconducting chalcogenide glasses is the presence of localized states in the mobility gap due to the lack of long-range order (LRO) and intrinsic defects. The conductivity in crystalline chalcogenides^10^ and chalcogenide glasses^11^ is usually suggested to be p-type. The idea of p-type conduction is based on the asymmetry of the conduction band (formed of anti-bonding orbitals) and the valence band (constituted by non-bonding or lone-pair electrons). It was suggested that the degree of the disorder should have more consequence on anti-bonding states as compared to non-bonding states.^12^ Therefore, the range of localized tail states at the valence band edge is less in comparison to the localized states at the conduction band edge. As a consequence, the number of electrons excited over the conduction band mobility edge is less than the number of holes excited below the valence band mobility edge. This is probably one of the reasons that chalcogenide glasses show p-type conductivity. Thus, the knowledge of the transport mechanism and estimation of the density of charged defect states is a significant study to understand the complete picture of electric conduction in chalcogen rich materials.

Dielectric and a.c. conduction properties of PVC gels, polymers, organic semiconductors and semiconducting glasses are considered as a significant tool for better understanding of the transport mechanism^8,13–15^ and novel phenomenon^9^ in such materials. Mott, Elliott and Davis proposed several band models in a series of papers for the explanation of the dielectric behavior and electronic structure of non-crystalline soft materials.^16^ Dielectric relaxation studies are important to understand the nature and the origin of loss, which in turn may be useful in the determination of the structure and defects in such solids.^17,18^ The dielectric dispersion is not expected in pure glassy selenium at low frequencies,^19,20^ as these materials are covalently bonded solids. However, the past measurements done by our and other groups^21,22^ indicate that the dielectric dispersion does exist in these glasses even at low frequencies. The origin and the nature of dielectric losses in these materials has, therefore, become a matter of curiosity. Investigations of the frequency and temperature dependence of the a.c. conductivity of chalcogenide glasses are also proved significant tools for understanding the nature of conduction mechanism in these materials.^17,18^ Therefore, we have synthesized novel third-generation multi-component glasses of SeTeSnM (where M is metallic impurity) by incorporating Cd, In and Sb as chemical modifier M in second generation SeTeSn glass. Further, we have studied the dependence of dielectric constant, loss, a.c. conductivity and the related parameters on temperature/frequency. These new studies are directly important to understand the “metallic-induced consequence” of dielectric relaxation and thermally assisted a.c. conduction. In past five decades, lots of work is done for establishing the glass forming regions on binary and ternary glasses, but no sufficient literature is available for quaternary glasses. Thus, we have started to incorporate such elements in Se–Te–Sn system; that show excellent glass forming tendency (GFT) and high thermal stability (TS) with Se–Te–Sn system so that it may be used as potential candidate for the optical memory applications.^2^

Efforts have been made by various workers frequently to investigate the compositional effects of a particular foreign element on dielectric and a.c. conducting parameters of all the three generations of these materials.^17–22^ To the best of our knowledge, no serious attempts have been done to make inquiries: what happens if we replace the doping element at fixed composition instead of inverse but the usual trend. Thus, this report of third generation glasses, where the composition is fixed and modifications have been analyzed by alteration of foreign atoms.

## 2. Experimental

### 2.1 Synthesis route

The exact proportions of high purity (99.999%) elements (purchased from Alfa Aesar), in accordance with their atomic percentages, were weighed using an electronic balance with the least count of 10^−4^ g. The materials were then sealed in evacuated (∼10^−6^ torr) quartz ampoules having length ∼5 cm and internal diameter ∼8 mm. The ampoules containing the constituent elements were heated to an appropriate temperature (∼650 °C) in a muffle furnace whose temperature was raised slowly at a rate of 3–4 °C per minute.

**Fig. 1:**
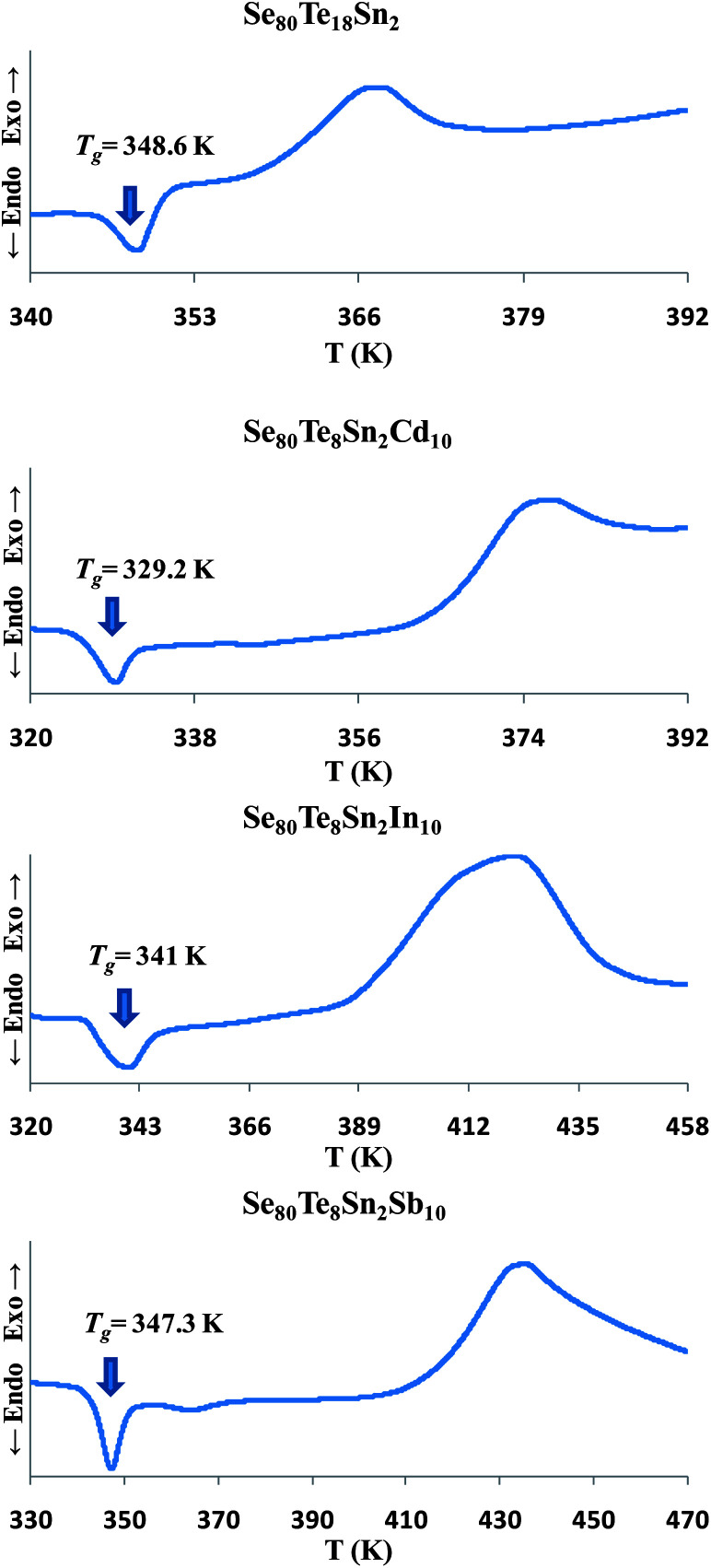
DSC scans of all samples showing their glass transition temperatures.

The thermal characterization was investigated using a differential scanning calorimeter (T.A. Instruments, USA; Model: Auto Q20) under almost identical conditions by using same heating rate (10 K min^−1^) and nitrogen as purge gas. Tzero aluminum pans and lids were used to seal the samples in powder form in DSC cell. The accuracy of the heat flow in DSC was ±0.01 mW and the temperature precision as determined by the microprocessor of the thermal analyzer was ±0.1 K. Fig. 1 shows the Differential Scanning Calorimetric (DSC) scans of all samples for confirmation of the glassy nature through the occurrence of sharp endothermic peaks in glass transition region before the appearance of exothermic peaks in crystallization region.

### 2.2 Dielectric and a.c. conductivity measurements

Electrical measurements were investigated in the audio frequency range (0.5 kHz–1 MHz) and temperature range 300–345 K for observing the appreciable and significant dielectric relaxation and a.c. conduction mechanism. The complex dielectric function of a material is represented by two parts. Its real part is known as dielectric constant (*ε*′) while its imaginary part is called dielectric loss (*ε*′′). The experimental values of capacitance and dissipation factor measured from LCR meter were used to evaluate *ε*′ and *ε*′′.

In chalcogenide glasses, the temperature/frequency dependence of dielectric constant (*ε*′) and dielectric loss (*ε*′′) are precise in the temperature range near glass transition.^17–22^ At temperatures lower than room temperature, *ε*′ is nearly constant and *ε*′′ is negligibly small. Above room temperature, *ε*′ and *ε*′′ are increased appreciably with temperature. Therefore, the present measurements have been made in the temperature region below the onset of glass transition region where dielectric dispersion is quite appreciable.

For measurements of dielectric constant and dielectric loss, all the glassy alloys were ground to a very fine powder and pellets (diameter ∼ 10 mm and thickness ∼ 1 mm) were obtained by compressing the powder in a die at a load of 5 tons. The pellets were coated with indium film to ensure good electrical contact between the sample and the electrodes. A specially designed metallic sample holder has been used for dielectric measurements. The temperature measurement was facilitated by a copper-constantan thermocouple mounted very near to the sample. An O-ring was present between the two parts of the sample holder for suitable evacuation inside the chamber. A vacuum of ∼10^−3^ torr was maintained over the entire temperature range from room temperature to near the glass transition region. Dielectric measurements were made using a digital LCR meter (Wayne Kerr Electronics, Model: 4100).

For accurate measurements and convenience, the sample is taken in cylindrical geometry. This can be achieved by pressing the powdered sample to prepare pellets since as-prepared materials cannot be shaped directly in perfect cylindrical geometry due to brittleness. Also, we preferred to experiment on the pellet rather than the bulk as macroscopic effects (gas bubbles, *etc.*) may appear in the bulk during preparation. It is already observed (both theoretically and experimentally) by Goyal *et al.*^19^ that bulk ingots and compressed pellets exhibit similar dielectric behavior in chalcogenide glasses.

## 3. Theoretical formalism to determine electrical parameters

Knowing the experimental values of the parallel capacitance and dissipation factor, the dielectric constant *ε*′ and dielectric values *ε*′′ can be calculated by using following relations:

1 *ε*′ = *C*/*C*_0_

2 *ε*′′ = *ε*′*D*

here *C* and *C*_0_ in eqn (1) represent the capacitance values for material and free space respectively. Further, *D* in eqn (2) represents the dissipation factor.

According to the model proposed by Guintini *et al.*,^23^ the dielectric loss (*ε*′′) at a particular frequency, in the temperature range where dielectric dispersion occurs, is expressed by the following relation:

3 *ε*_2_ = (*ε*_s_ − *ε*_∞_)2π^2^*N*(*ne*^2^/*ε*_s_)^3^*k*_B_*Tτ*^m^_0_*W*_m_^−4^*ω*^m^

here, *n* is the number of electrons that hop, *N* is the concentration of localized sites, *ε*_s_ and *ε*_∞_ are the static and optical dielectric constants, respectively, *W*_m_ is the energy required to move the electron from a site to infinity. Further, *e* is the electronic charge, *k*_B_ the Boltzmann's constant and *T* the absolute temperature.

Here *m* is the power of angular frequency (*ω*) and it is given by:4
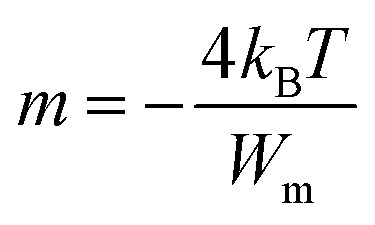


The value of a.c. conductivity is calculated by the relation:

5 *σ*_ac_(*ω*) = *ωε*′′*ε*_0_

here *ε*_0_ is the permittivity of free space and *ω* is the applied field angular frequency.

## 4. Results and discussion

**Fig. 2:**
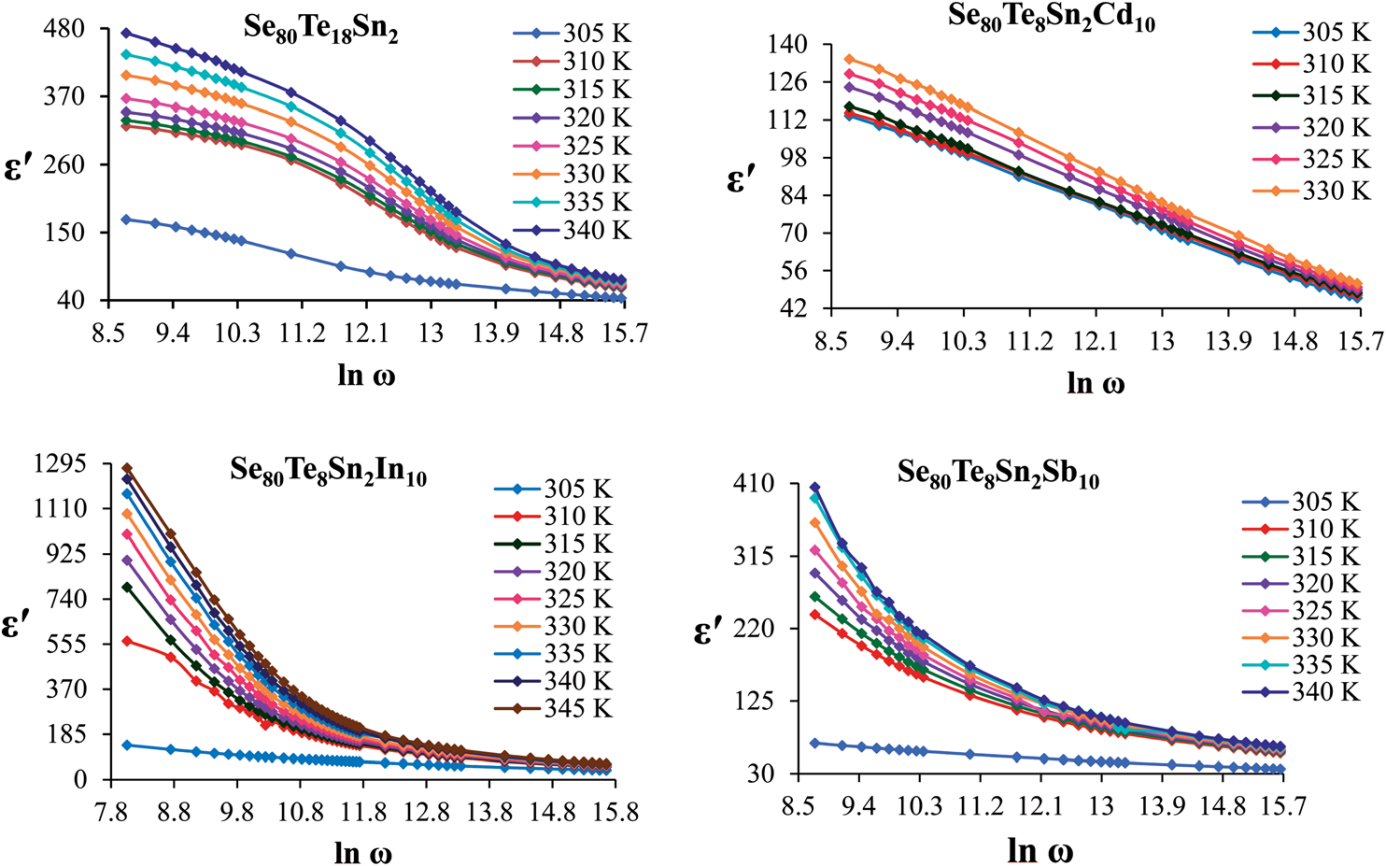
Plots of *ε*′ *versus* ln *ω* for glassy Se_80_Te_18_Sn_2_ and Se_80_Te_8_Sn_2_M_10_ alloys.

The frequency dependence of dielectric constant for the present glassy SeTeSnM system at different temperatures is shown in Fig. 2. A strong frequency dispersion of *ε*′ is observed in the low-frequency region followed by almost frequency independent behavior beyond ∼10 kHz for all temperatures. It is revealed from these plots that the values of *ε*′ increase with increase in temperature at all frequencies for the present glassy system. This increment is found different for different frequencies. The observed large values of *ε*′ in the lower frequency range can be explained on the basis of the Maxwell–Wagner model.^24^ According to Koops's theory,^25^ the solid is assumed to be formed of well-conducting grains separated by highly resistive thin layers and grain boundaries. As a result of the applied signal to the material, a space charge polarization is built up at the grain boundaries. The induced space charge polarization is restricted by the available free charges on the grain boundary and the conductivity of the sample. Further, Koops's model suggests that the major contribution of the dielectric constant at low frequency comes from the grain boundaries, which have a high dielectric constant. On the other hand, at high frequency, the dielectric behavior of the material is dominated by the grains which have a small dielectric constant. The decrease of *ε*′ with the increase in frequency may be recognized by the electrical relaxation processes.

**Fig. 3:**
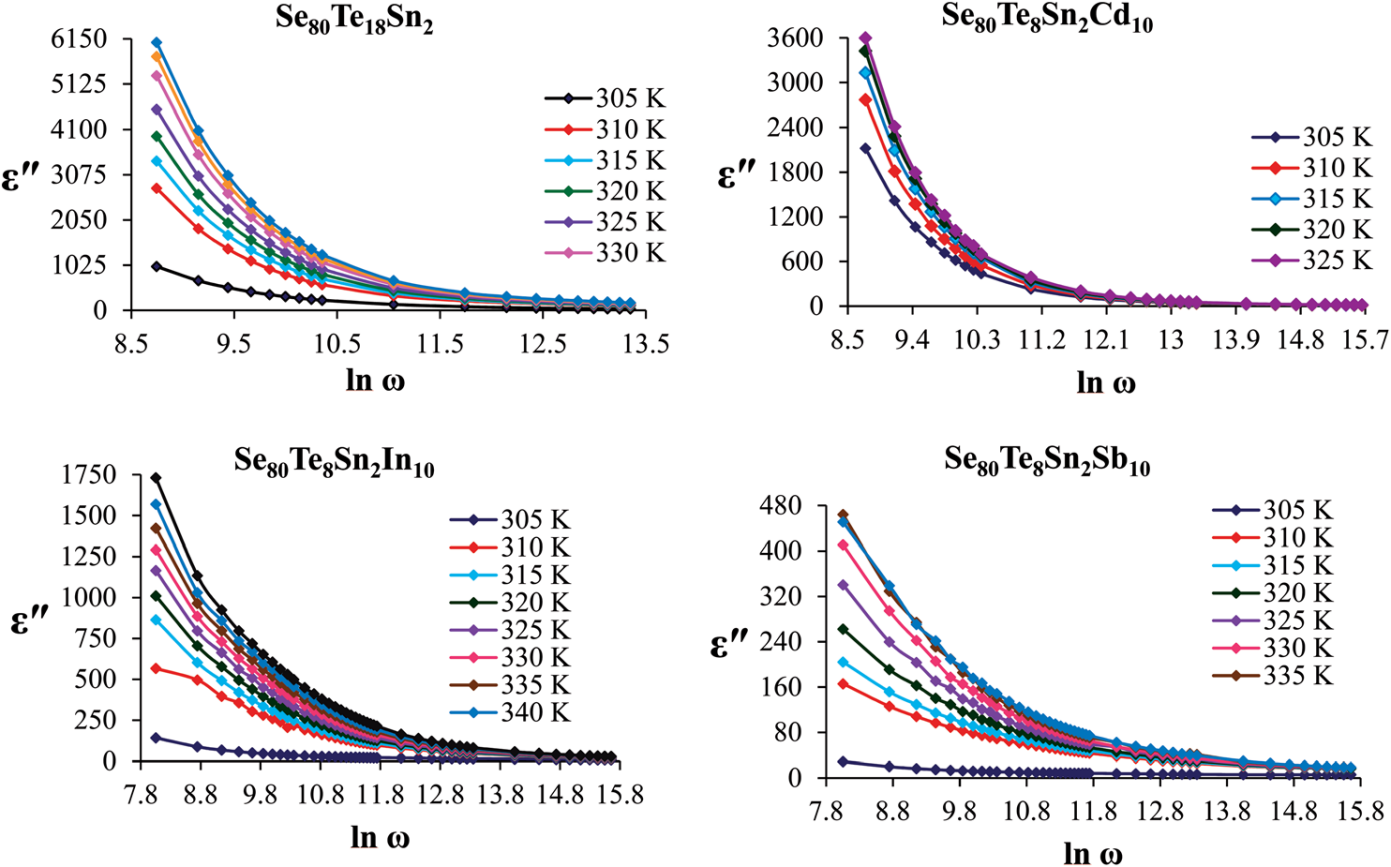
Plots of *ε*′′ *versus* ln *ω* for glassy Se_80_Te_18_Sn_2_ and Se_80_Te_8_Sn_2_M_10_ alloys.

The frequency dependence of the imaginary component of the dielectric function, *i.e.*, dielectric loss *ε*′′ for the present glassy system in the aforesaid frequency and temperature region is represented in Fig. 3. It is obvious from these plots that the dielectric loss also has large values in the low-frequency range that are decreased with increasing frequency. At higher frequencies, *ε*′′ has no remarkable changes at all temperatures. In the low-frequency region, which corresponds to high resistivity due to the main cause of the grain boundaries at lower frequencies, more energy is required for hopping between the levels. Therefore the energy loss is high at lower frequencies. In the high-frequency region, the less energy is required for electron movement between the levels and thus the energy loss is small. The inverse frequency dependence of the dielectric loss at low frequencies was evidence of the non-Debye type character of the overall relaxation. It is well known that in Debye-type relaxation, the loss factor should increase with increasing frequency.

**Fig. 4:**
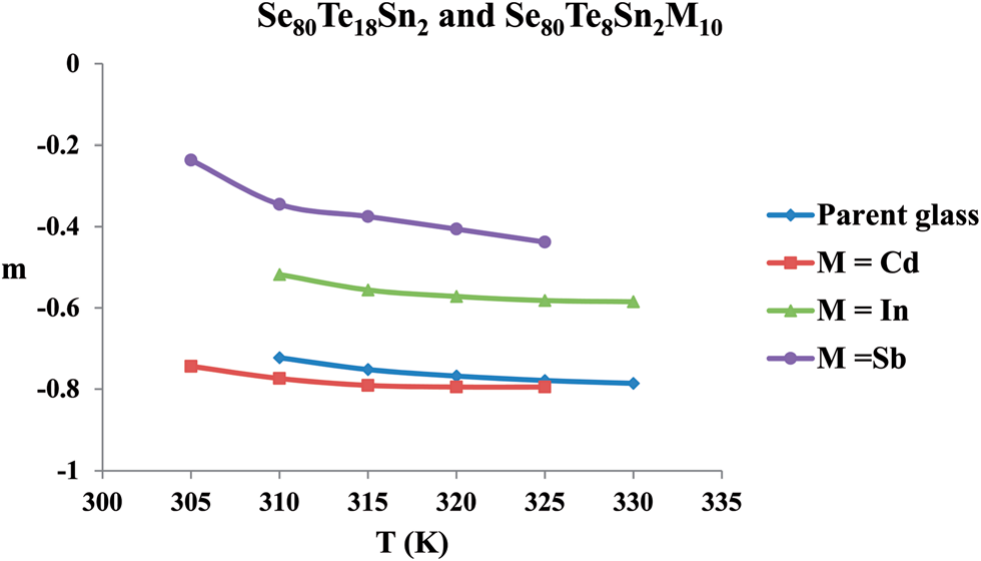
Temperature dependence of parameter *m* for glassy Se_80_Te_18_Sn_2_ and Se_80_Te_8_Sn_2_M_10_ alloys.

The frequency exponent (*m*) is obtained by least squares fitting of the experimental data and is plotted as a function of temperature in Fig. 4 for multi-component glasses. It is clear from these plots that the calculated values of the frequency exponent *m* are decreased with increasing temperature. This observation is consistent with Guintini^23^ theory which shows that the dielectric relaxation is related to the hopping of charge carriers over a potential barrier.

**Fig. 5:**
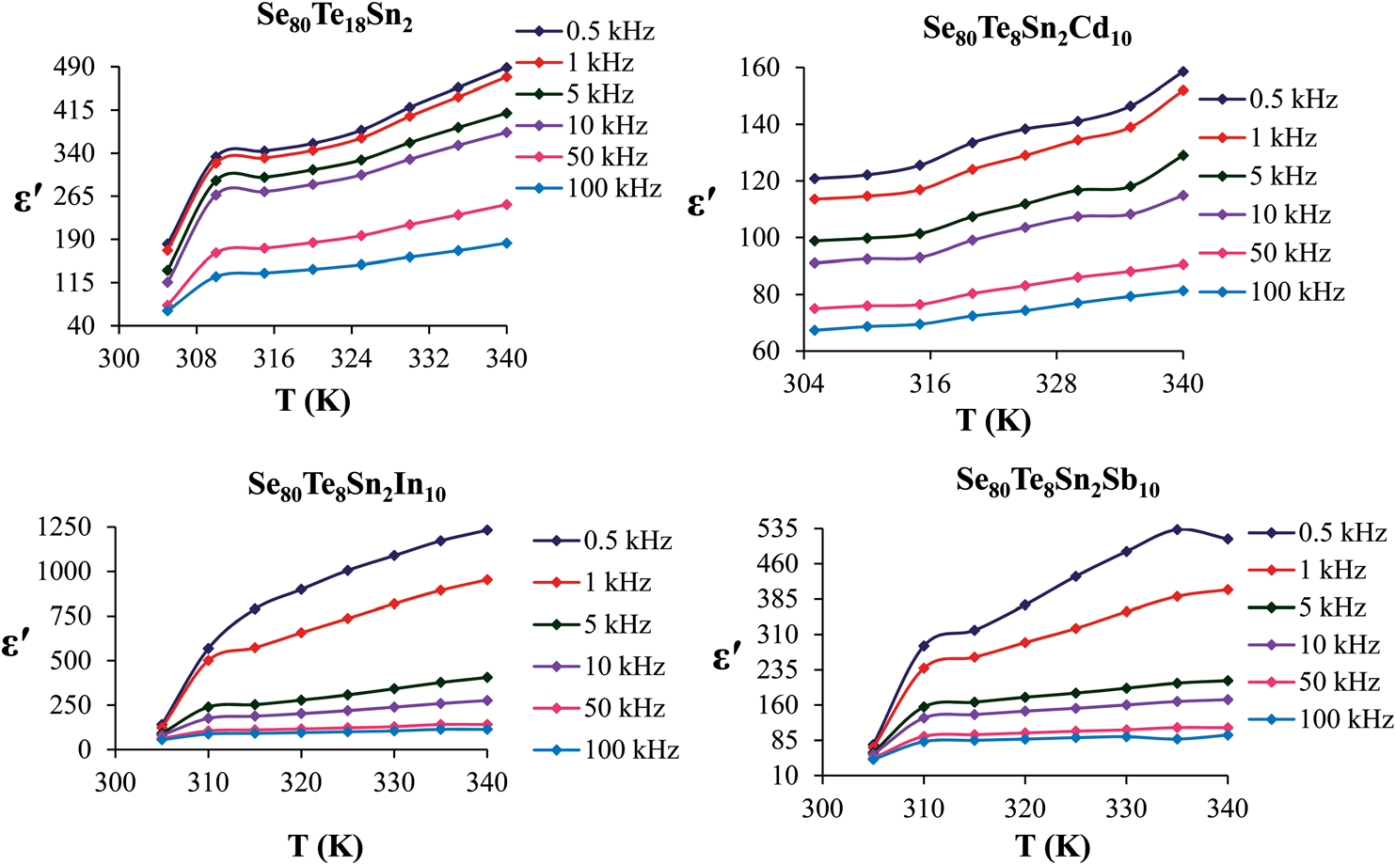
Temperature dependence of dielectric constant for glassy Se_80_Sn_2_Te_18_ and Se_80_Te_8_Sn_2_M_10_ alloys.

The temperature dependency of *ε*′ at various frequencies is represented in Fig. 5 for the present glassy system. These plots clearly indicate that *ε*′ increases with the increase in temperature at all frequencies. Further, it is also evident from these figures that *ε*′ shows strong temperature dependence in the low-frequency region. On the other hand, it exhibits weak temperature dependence in the high-frequency region. Such temperature dependence of *ε*′ can be explained in terms of orientational polarization which is associated with the thermal motion of molecules. At relatively low temperatures, the dipoles cannot orient themselves with respect to the track of the applied field. Consequently, their contribution to the polarization and the dielectric constant is weak. As the temperature rises, the bound charge carriers get sufficient excitation thermal energy and they become capable to follow the change in the external field more easily. This, in turn, enhances their involvement with the polarization leading to an increase in the dielectric constant of the sample.^26^

**Fig. 6:**
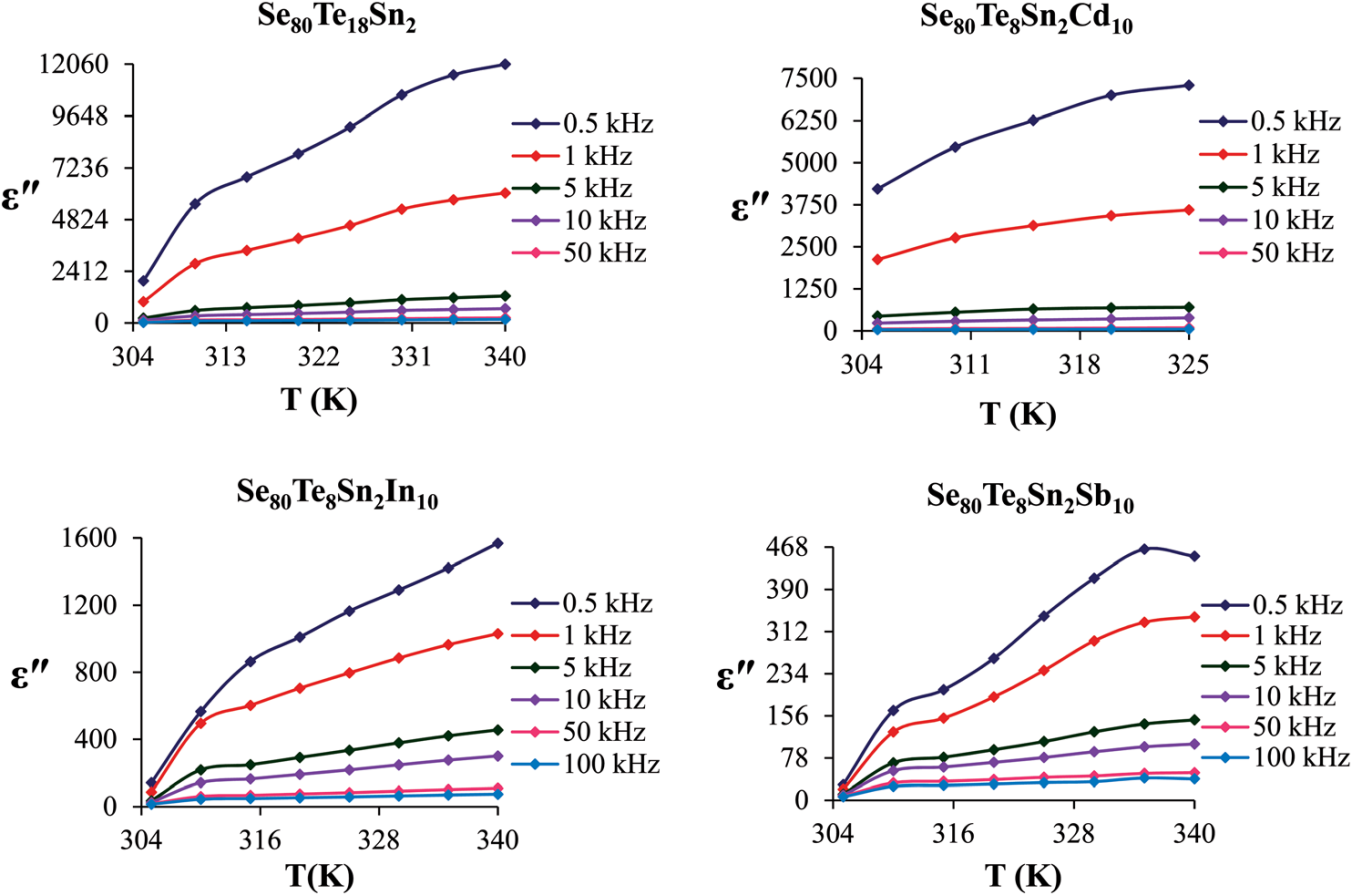
Temperature dependence of dielectric loss for glassy Se_80_Te_18_Sn_2_ and Se_80_Te_8_Sn_2_M_10_ alloys.

Fig. 6 shows the temperature dependence of *ε*′′ at various frequencies. It is obvious from these figures that *ε*′′ also increases with increasing temperature. Unlike the electronic and ionic polarization, the orientational polarization involves associative motions of molecular segments in a viscous medium and it depends on temperature. It is well known that the rise in temperature leads to a fall in viscosity of the medium. The effect of the drop in viscosity on the amount of the dielectric loss can be considered as an increase in the internal friction of matter when the dipole rotates through a unit angle. Interfacial or space charge polarization is an additional feature that should be recognized and accounted for the explanation of experimental outcomes. This type of polarization is due to the migration of electrons or ions over distances of macroscopic scale. Distinct from deformational polarization (electronic and ionic), the relaxation polarization (orientational and interfacial) needs a quite long time and dissipates electric energy that transforms into heat in a dielectric, *i.e.*, this energy causes dielectric loss. According to Stevels,^26^ the relaxation phenomenon divided in following three parts: (i) conduction loss, (ii) dipole loss and (iii) vibrational loss. The conduction loss has a minimum value at the low temperature because it is proportional to *σ*_ac_(*f*)/*f*. Since *σ*_ac_(*f*) increases with increasing temperature, therefore it causes the increase in conduction losses. Consequently, the value of *ε*′′ is increased with increase in temperature. The other two losses involve the migration of ions over large distances. This motion is the same as that occurring under direct current conditions. The ions jump over the highest barriers in the network. At low-temperature values, conduction, dipole and vibration losses have the minimum value. However at higher temperatures, as the temperature increases, the hopping process increases and since the conduction is due to hopping from one localized states to the another, thus the conduction increases and so the losses due to conduction also increases. Additionally, dipole and vibration losses also increase.

Structural studies of chalcogenide glasses are very important for better understanding of transport mechanisms. A.c. conductivity measurements have been widely used to investigate the nature of defect centers in disordered systems since it is assumed that they are responsible for this type of conduction. The general frequency behavior in this type of materials is of *Aω*^s^ type over a wide frequency range where exponent *s* is found to be temperature dependent and has a value ≤ 1. Various models have been formulated to explain this behavior and classical approach is to consider a.c. and d.c. conduction separately.

The a.c. conductivity in semiconductors has been interpreted in terms of the pair approximation. In this approximation, a pair consists of two localized states between which electronic carriers move back and forth with a particular relaxation time. In amorphous semiconductors, the localized states have been considered to be randomly distributed in the space, and pairs with various relaxation times exist. The a.c. conductivity is the sum of contributions from all the pairs.

A.c. conductivity *σ*_ac_(*ω*) can be modeled by a power law:^27^

**Fig. 7:**
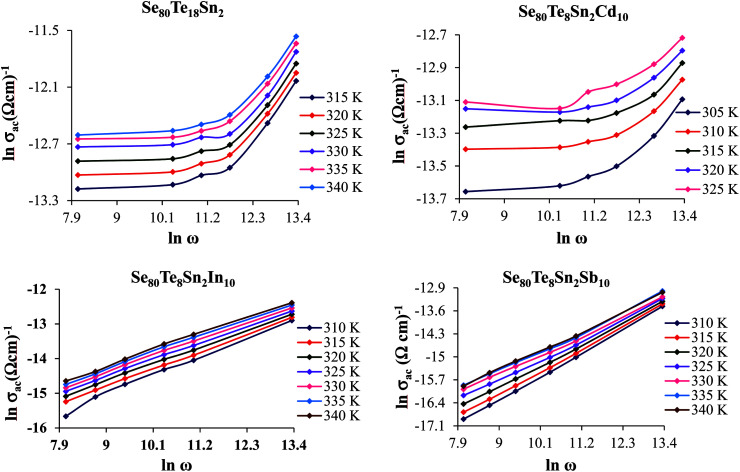
Plots of ln *σ*_ac_*vs.* ln *ω* for glassy Se_80_Te_18_Sn_2_ and Se_80_Te_8_Sn_2_M_10_ alloys at different temperatures.

**Fig. 8:**
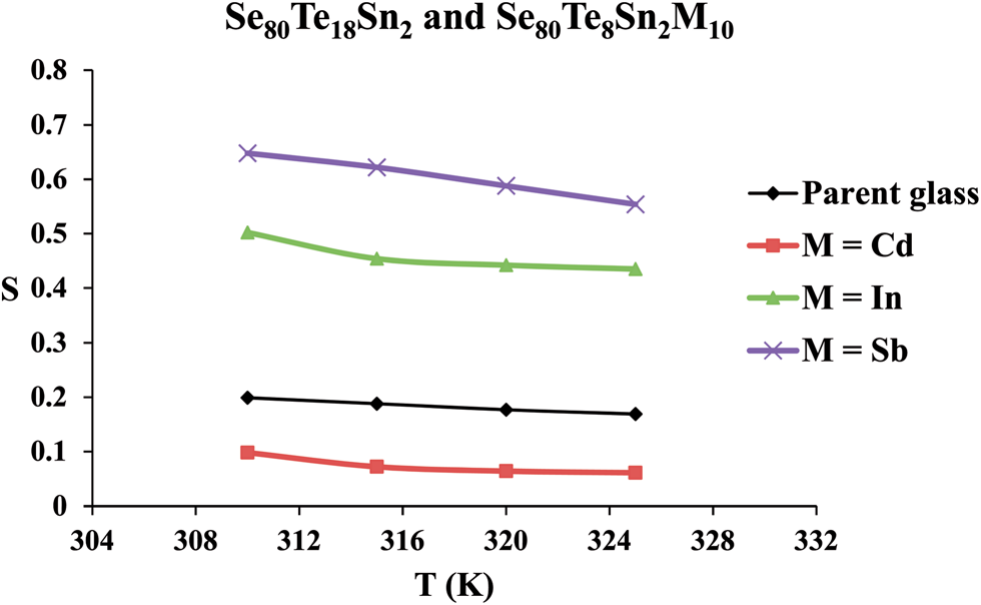
Temperature dependence of parameter *s* for glassy Se_80_Te_18_Sn_2_ and Se_80_Te_8_Sn_2_M_10_ alloys.

6 *σ*_ac_(*ω*) = *σ*_tot_(*ω*) − *σ*_dc_(*ω*) = *Aω*^s^

here *ω* is the angular frequency (*ω* = 2π*f*), *A* is a constant, *f* is the operating frequency and *s* is the frequency exponent parameter, which depends on temperature. Eqn (6) is frequently used for the study of frequency dependence of the a.c. conductivity during thermally activated a.c. conduction. It represents the degree of interaction between mobile ions and the environments surrounding them. Since the obtained d.c. conductivity *σ*_dc_ is much smaller than total electrical conductivity, therefore *σ*_dc_ can be ignored in eqn (6). Thus, *σ*_tot_(*ω*) is considered to be *σ*_ac_(*ω*). The frequency dependence of *σ*_ac_(*ω*) for all glassy alloys is calculated in the frequency range (0.5–100 kHz) at different values of temperature in the glass transition region. Fig. 7 shows the frequency dependence of a.c. conductivity *σ*_ac_(*ω*) for the glassy SeTeSnM system at different temperatures. It is clear from these figures that *σ*_ac_(*ω*) increases almost linearly with increasing frequency. Values of *s*(*T*) have been calculated from the slopes of the straight lines of these figures. The temperature dependence of exponent *s* is shown in Fig. 8 for glassy SeTeSnM systems. These plots demonstrate that exponent *s* is inversely proportional to temperature. The observed behavior of *s*(*T*) indicates that the correlated barrier hopping (CBH) is the possible conduction mechanism in the present glassy alloys.^27^

**Fig. 9:**
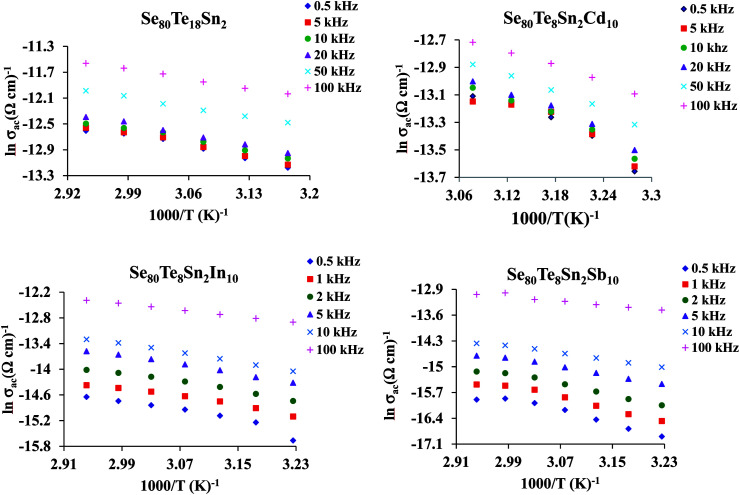
Plots of ln *σ*_ac_ against 1000/*T* at different frequencies for glassy Se_80_Te_18_Sn_2_ and Se_80_Te_8_Sn_2_M_10_ alloys.

The plots of *σ*_ac_ against 1000/*T* for all glasses at different frequencies are shown in Fig. 9 for the glassy SeTeSnM system. From these plots, one can see that *σ*_ac_ increases with increase in frequency. It is also clear from these figures that *σ*_ac_ increases linearly with decreasing the reciprocal of absolute temperature. This clearly indicates that *σ*_ac_ has Arrhenian temperature dependence. Arrhenian temperature dependence is generally observed in a variety of physical and chemical quantities during studies of different kinds.^28–32^

In other words, the a.c. conduction is a thermally activated process due to hopping in the localized states between the gap or band tails. From these plots, it is clear that the variation of a.c. conductivity with temperature can approximately be expressed by well known Arrhenius relation in the intermediate temperature range as:^16,33^7
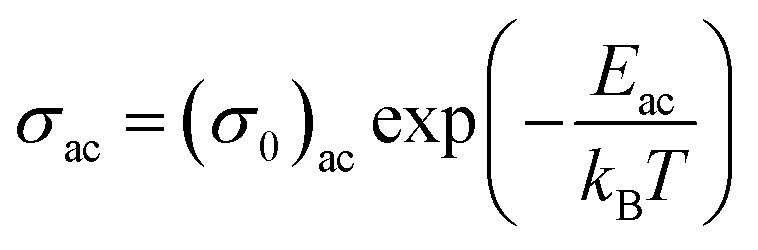
here (*σ*_0_)_ac_ is the pre-exponential factor of a.c. conduction and *E*_ac_ is the corresponding activation energy. The values of *E*_ac_ at different frequencies are given in Table 1 for the present glassy system. From this table, it is clear that the activation energy decreases with increasing frequency. This decrease may be attributed to the increase of the applied frequency which enhances the electronic jump between the localized states.

**Table 1:** Frequency dependence of activation energy of a.c. conduction *E*_ac_ for glassy Se_80_Te_18_Sn_2_ and Se_80_Te_8_Sn_2_M_10_ alloys

Frequency (kHz)	*E*_ac_ (eV)			
	Se_80_Te_18_Sn_2_	Se_80_Te_8_Sn_2_Cd_10_	Se_80_Te_8_Sn_2_In_10_	Se_80_Te_8_Sn_2_Sb_10_
0.5	0.25	0.23	0.31	0.33
10	0.22	0.21	0.24	0.20
20	0.20	0.20	0.22	0.17
100	0.18	0.16	0.16	0.14

Like other functional materials,^34–36^ chalcogenide glasses also follow correlated barrier hopping model and therefore the advantages of a.c. measurements are that they permit investigations of time-dependent properties such as polaron hopping and information of other processes occurring in the material. Such measurement also helps to estimate the density of charged defect states.^37^

As mentioned earlier, the a.c. conduction in chalcogenide glasses can be explained in terms of both single polaron hopping and bi-polaron hopping. With this point of view, the experimental data have been fitted according to CBH model. The fitting has been applied to the product *NN*_p_ of *N* and *N*_p_ for achieving a satisfactory agreement between the theoretical and experimental curves of ln *σ*_ac_(*ω*) *versus* 1000/*T*. The fitting has been done at a frequency of 10 kHz. The values of *W*_m_ for different alloys are calculated using temperature dependence of exponent *s*:^27^8
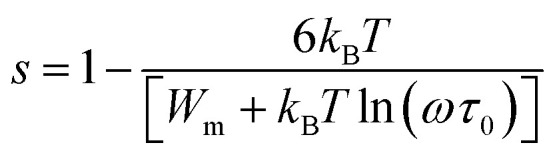
here *τ*_0_ is characteristics relaxation time which is of the order of a period of atomic vibration (∼10^−12^ s).

**Fig. 10:**
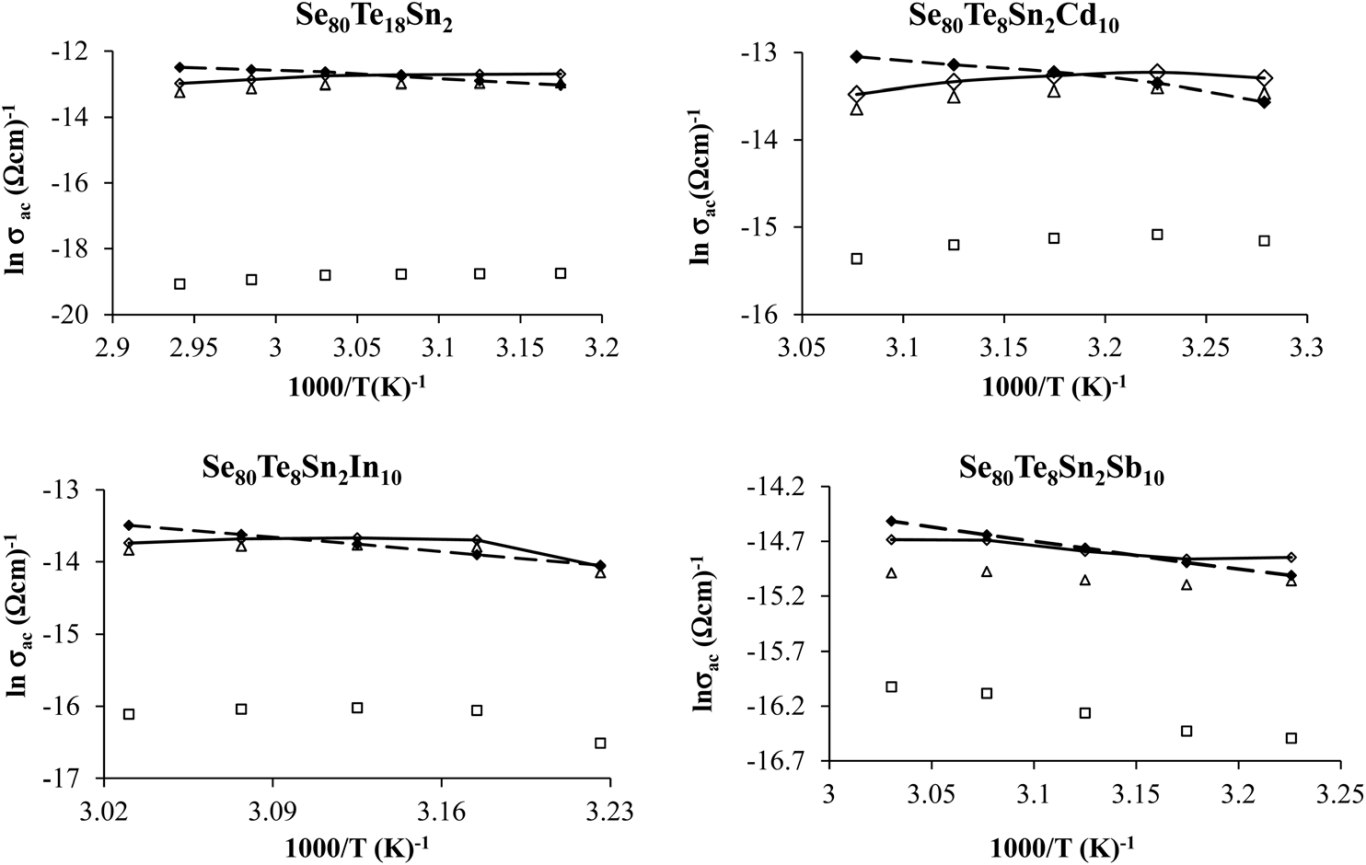
Fitting of theoretical and experimental results of a.c. conductivity for glassy Se_80_Te_18_Sn_2_ and Se_80_Te_8_Sn_2_M_10_ alloys.

Fig. 10 shows the contributions of single-polaron and bi-polaron hopping and their sum to the thermally assisted a.c. conduction at a frequency of 10 kHz. In these plots, the points shown by symbols 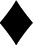, 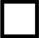, 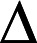 and 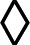 stand for experimental a.c. conductivity (*σ*_ac_)_exp_, the contribution of single polaron hopping to a.c. conductivity (*σ*_ac_)_si_, contribution of bi-polaron hopping to a.c. conductivity (*σ*_ac_)_bi_ and theoretical a.c. conductivity (*σ*_ac_)_theor_ respectively. As seen from these figures, theoretical curves are in good agreement with the experimental results. It is also clear from these figures that bi-polaron contribution is dominant over single polaron for present alloys. The density of charged defect states *N* is evaluated from the values of *NN*_p_ (where *N*_p_ = *N*/2). The values of *N* are given in Table 2.

**Table 2:** Density of charged defect states for both glassy systems

Sample	*N* (cm)^−3^	LP
Se_80_Te_18_Sn_2_	4.0 × 10^22^	3.92
Se_80_Te_8_Sn_2_Cd_10_	7.9 × 10^20^	3.32
Se_80_Te_8_Sn_2_In_10_	1.3 × 10^22^	3.52
Se_80_Te_8_Sn_2_Sb_10_	1.6 × 10^22^	3.67

**Fig. 11:**
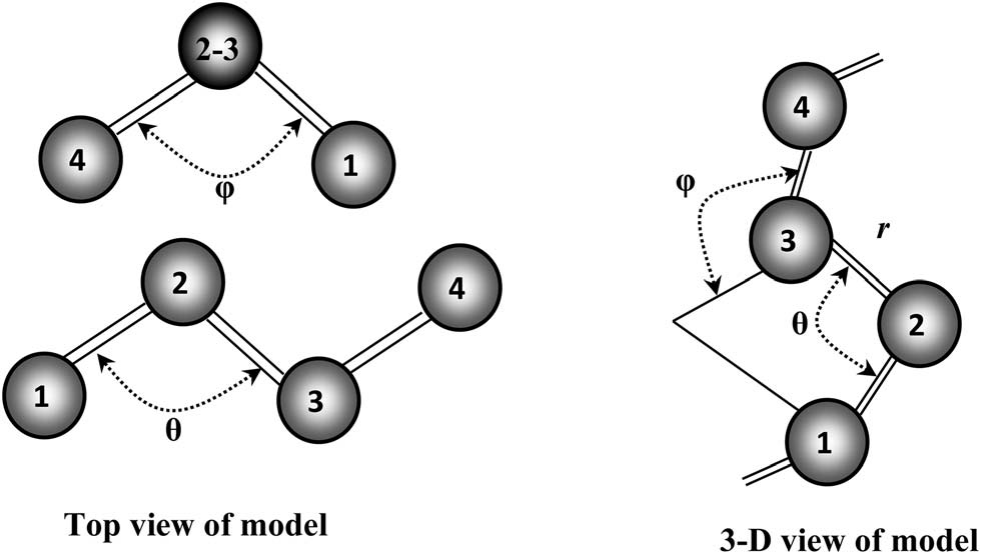
Random chain model of Se chain molecule.

Our recent structural and Raman spectroscopic investigations of Se and Se-rich As_*x*_Se_1−*x*_ amorphous thin films suggest that all the atoms are in a twofold coordinated chain structure and not random. This so-called “random chain model” defines the angle between two adjacent bonding planes as a dihedral angle *φ* (see Fig. 11). The magnitude of *φ* remains constant with random variation in sign.^38^ The breakup of the Se_8_ ring structure takes place due to the incorporation of Te in Se–Te glasses. Consequently, the chain fraction slightly increases with some reduction in the chain length. Further incorporation of foreign elements in glass matrix of Se–Te also affects its molecular structure^38^ by the formation of cross-linking structures.

Keeping in mind the above model, the increase in the value of the density of localized states after incorporating the metallic additives M in parent Se_80_Te_18_Sn_2_ alloy can be explained to some extent in terms of average electro-negativity *χ* of quaternary Se_80_Te_8_Sn_2_M_10_ alloys. In the present case, we have added Cd, In and Sb in parent alloys at cost of Te. Defect states are assumed to be created because of the difference between electro-negativity *ξ*_M_ of the metallic additive M and that of the replaced element (here *ξ*_Te_ for Te) of parent alloy. It is, therefore, reasonable to expect that the difference of the density of defect states will be more where the electro-negativity difference (*ξ*_M_ ~ *ξ*_Te_) for the quaternary alloy is large. Since defect states are assumed to be created because of the formation of alloys from constituent atoms. The values of *χ* for replaced element Te and incorporated atoms M are given in Table 3.^39^ From this table, it is clear that increasing sequence (Δ*ξ*)_M=Sb_ < (Δ*ξ*)_M=In_ < (Δ*ξ*)_M=Cd_ of Δ*ξ* (=*ξ*_M_ − *ξ*_Te_) is same as the increasing sequence of the difference of the density of charged defect states *N* between the parent ternary alloy and the quaternary alloys.

**Table 3:** Values of average electro-negativity for the elements Te, Cd, In and Sb

Element	*χ*
Te	2.618
Cd	1.978
In	2.138
Sb	2.458

**Fig. 12:**
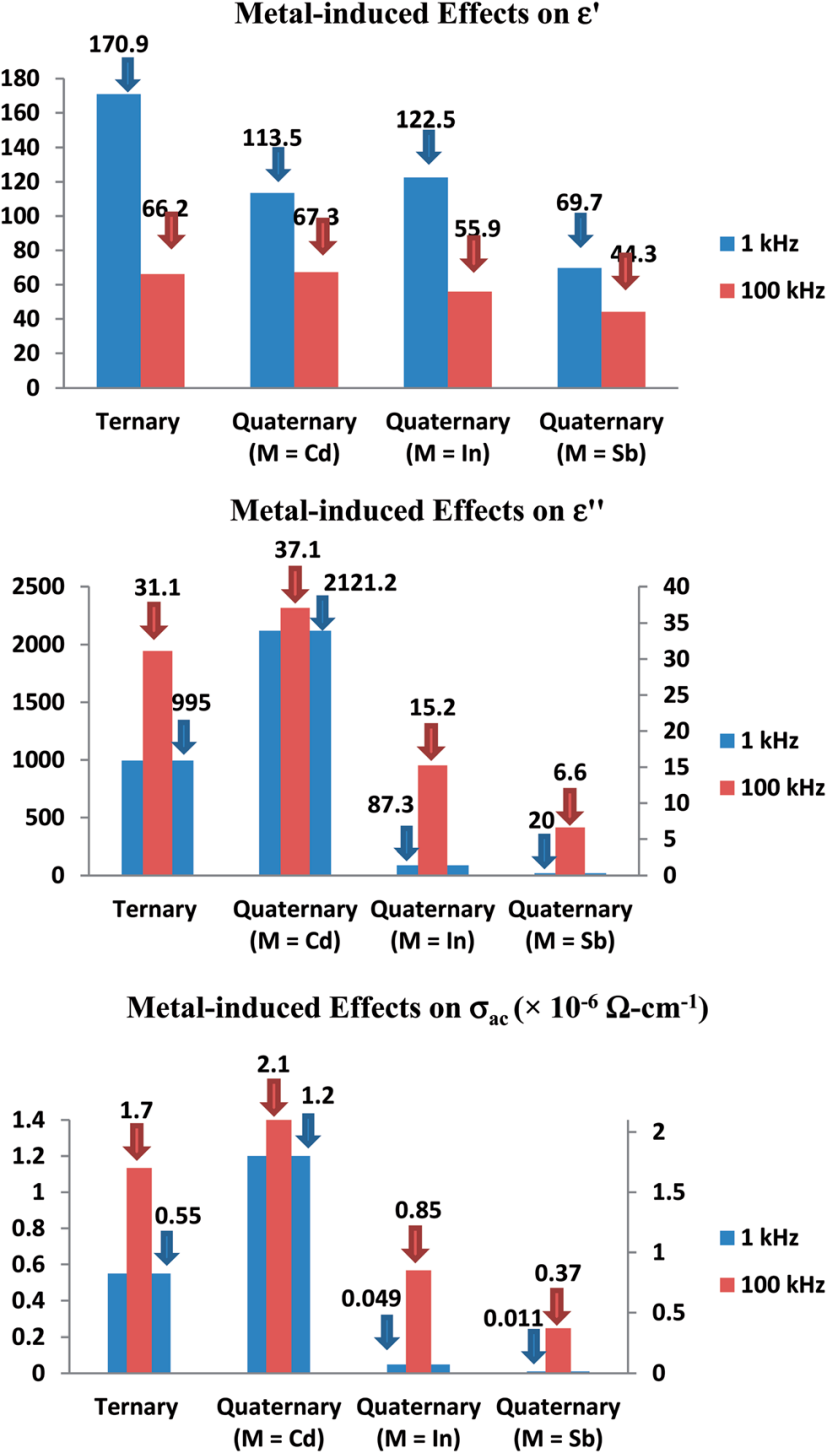
Comparison of metal-induced effects in dielectric constant, loss and a.c. conductivity of parent glass.

The comparison of the effect of the fourth component (Cd, In or Sb) on the dielectric properties and a.c. conductivity is shown in Fig. 12 in terms of plots at room temperature (305 K) at two different frequencies. From these plots, one can see the significant variation in dielectric constant, loss and a.c. conductivity of parent ternary Se_80_Te_18_Sn_2_ glass after incorporation of Cd, In or Sb. Similar results have been observed at other temperatures and frequencies (not shown here). However, the variation in different properties for different additives is not systematic and it is highly diverse in nature.

The structure of glassy Se and the outcome of alloying Te into glassy Se have been observed by different research groups.^40–43^ They found the presence of long polymeric chains and rings of Se atoms in glassy Se, which are dissociated by the addition of Te in glassy Se. The successive incorporations of Sn in Se–Te glass matrix and then Cd, In and Sb in Se–Te–Sn glass matrix causes the cross-linking of Sn and Cd, In and Sb in long polymeric Se chains and Se–Te mixed chains. Thus, the dielectric constant, loss and a.c. conductivity in parent glass are changed appreciably after the addition of the fourth element. Further, the occurrence of a.c. conduction takes place from different localized states created in the gap or its tails of present selenium-rich glass due to addition of Te, Sn and Cd. Thus, the dielectric constant, loss and a.c. conductivity changes noticeably in quaternary Se_80_Te_8_Sn_2_M_10_ (M = Cd, In and Sb) glasses due to variation in the number of dangling bonds in the glassy network of the parent ternary Se_80_Te_18_Sn_2_ alloy.

These structural defects are not steady in the natural state. Such defects become moderately stable when polarized to positively charged and negatively charged defect states. The density of these native charged defects can be deduced from the mass action law as follows using the notation of Kastner *et al.*^44,45^

9 2C^0^_2_ → C_3_^+^ + C_1_^−^

The charged states were represented by this group as C_1_^−^ and C_3_^+^. Here, the symbol C is labeled as chalcogenide and subscript indicates the atomic coordination and C^0^_2_ stands for neutral center keeping in mind that an extra electron placed on C^+^_3_ is shared equally between the three bonds of the atom, which therefore remains three-fold coordinated.

In the case of pure Se, structural defects, such as dangling atoms occur because of its non-crystalline character. These structural defects are not stable in the natural state. They are fairly stable when polarized to positively charged and negatively charged defect states.

When we use the mass action law in eqn (9), we see that:10
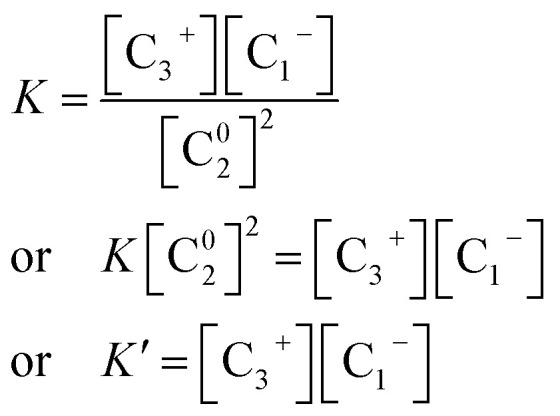
here *K*′ = *K*[C^0^_2_]^2^, because [C^0^_2_] is a constant.

The neutral condition is

11 [C_3_^+^] = [C_1_^−^]

These structural defects are believed to form localized energy levels in the band gap of Se and to act is deep traps for carrier transport.

In ternary Se_80_Te_18_Sn_2_ alloy, all Sn atoms are probably mixed in Se atomic chains due to lower concentration, but some of Te atoms would act as ionized impurities due to the higher concentration since the electron affinity of Te is lower than that of Se.

In case of quaternary alloys *i.e.*, the ternary alloy having incorporation of foreign additives M, some Te atoms act as positively charged impurities and metallic atoms also act as positively charged impurities. Therefore, the above neutral condition represented by eqn (11) takes the form:

12 [C_3_^+^] + [Te^+^] + [M^+^] = [C1^−^]

When we insert eqn (12) into (10), we obtain:13
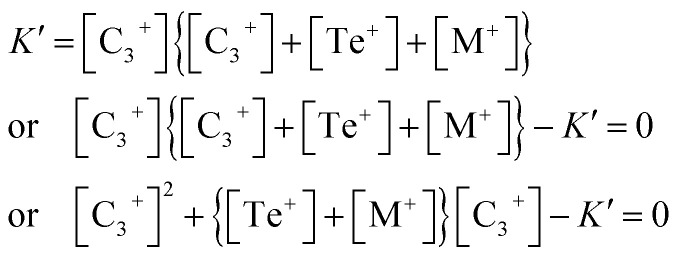


This is a quadratic equation in [C_3_^+^], whose solution is:14
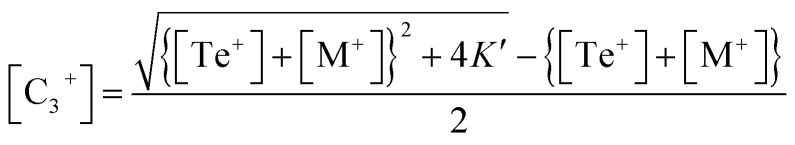


Now eliminating [C_3_^+^] from eqn (12) and (10), we have:15
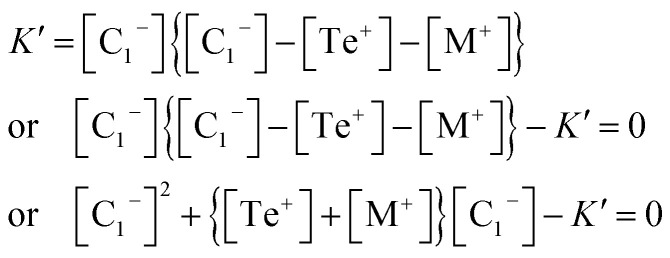


This is a quadratic equation in [C_1_^−^], whose solution is:16
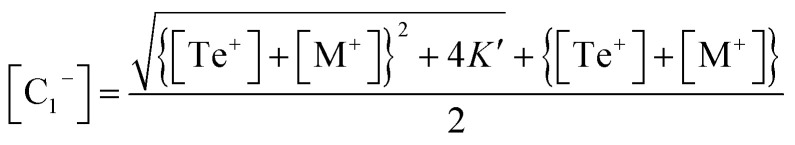


From eqn (14) and (16), it is can be seen that after incorporating the metallic impurities M (M = Cd, In and Sb) into parent Se_80_Te_18_Sn_2_ glass, the density of structural defects is changed.

Chalcogenide glasses are frequently known as lone pair semiconductors.^46,47^ Lone pair electrons represent the pairs of valence electrons that do not participate in forming covalent bonds with the neighbouring atoms. The density of defect states in the band tails related to the dielectric behavior and conduction of chalcogenide glasses mark their strong dependency on lone pair electrons and.^46,47^ The lone pair orbits have higher energy than the bonding states and hence occupy the top of the valence band. Interactions between lone pair electrons with their local environment and different atoms result in localized states in the band tails.^44^

**Fig. 13:**
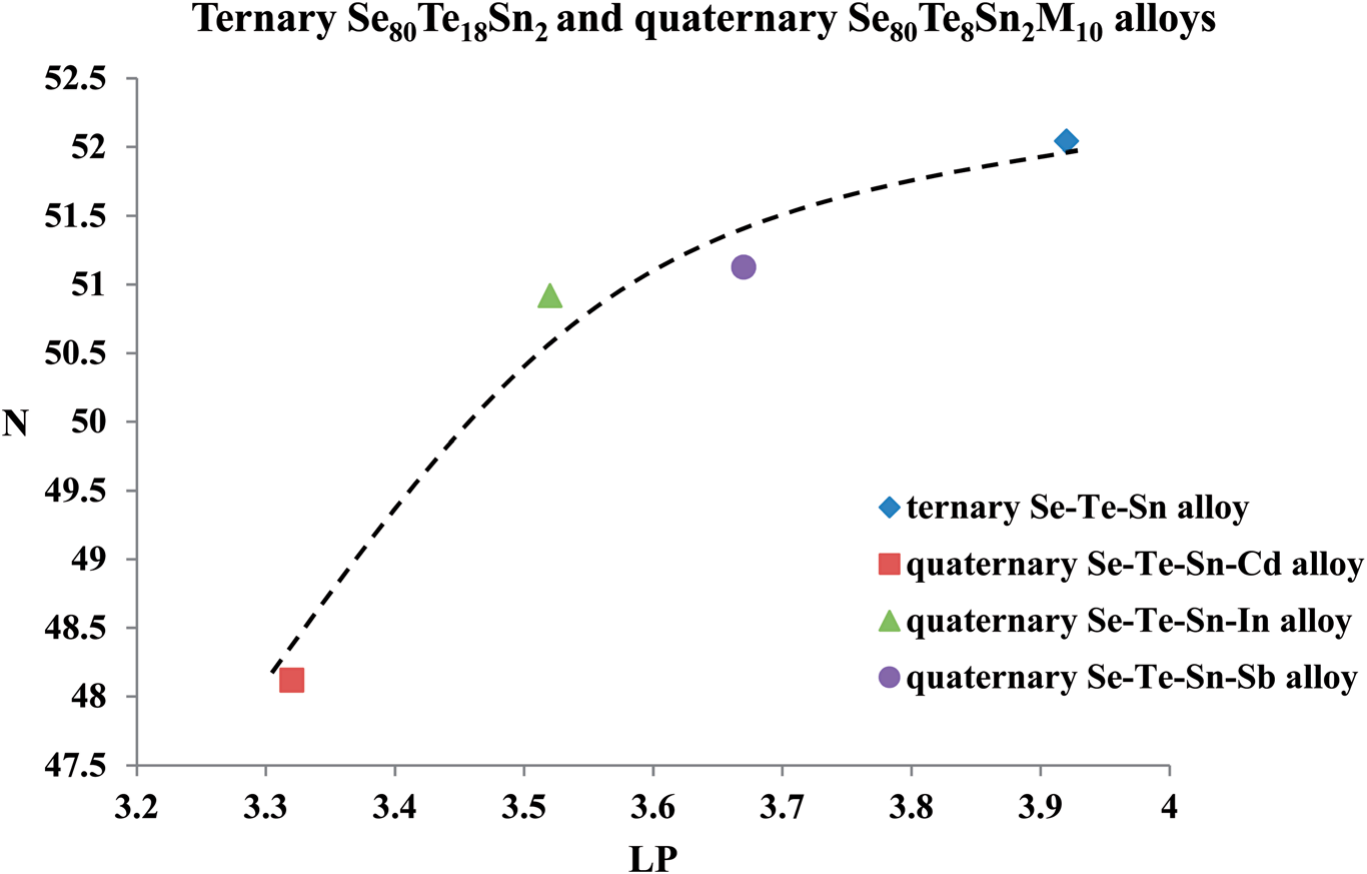
Variation of density of charged defect states *N* with number of lone-pair electrons LP (line is guided to eyes).

The formula for the determination of lone-pair electrons (LP) is:

17 LP = *V* − 〈*r*〉

here LP is the number of lone-pair electrons, *V* is the valence electron which is equal to unshared lone-pair electron and 〈*r*〉 is the average coordination number. The evaluated values of LP for multi-component glasses are also given in Table 2. One can see that the values of the density of charged defects states (*N*) in ternary Se_80_Te_18_M_10_ parent glass and quaternary Se_80_Te_8_Sn_2_M_10_ alloys varies according to the variation in values of LP [see Fig. 13].

Recently, we have observed that when the additives Cd, In and Sb are incorporated in the present ternary parent glass, they affects its equilibrium liquid-specific heat and glass specific heat noticeably. Thus, the present results of metal-induced effects of Cd, In and Sb on electrical properties are in consistent with that have been observed in the thermodynamic properties.^48^

## 5. Conclusions

The dielectric constant and loss both are decreased in low-frequency range, while there is no remarkable change in both parameters in higher-frequency range for all the studied glassy alloys. Both dielectric constant and loss and a.c. conductivity are changed significantly after incorporation of cadmium, indium and antimony in ternary Se_80_Te_18_Sn_2_ parent glass. Due to the enhancement of complexity of structure for the glassy Se–Te–Sn–M system (having four elemental components), the explanation of such anomalous behavior is not possible at this stage. The frequency dependence of a.c. conductivity obeys the power law. Further, the a.c. conductivity *σ*_ac_ shows Arrhenius temperature dependence. The temperature dependence of frequency exponent *s* and its lower values (<1) verifies the applicability of the CBH model in present alloys.

The results regarding the metal-induced effects of Cd, In and Sb on dielectric properties are explained in terms of the significant variation in the number of dangling bonds as suggested by Kasner's model. The effect of incorporation of Cd, In and Sb on the density of charged defect states in quaternary Se_80_Te_8_Sn_2_M_10_ alloys is explained in terms of electro-negativity difference (*ξ*_M_ − *ξ*_Te_) and the number of lone-pair electrons. As we know that the dielectric relaxation and thermally assisted a.c. conduction play a key role for deciding the usefulness of chalcogenide glasses in various applications like dielectric mirrors, capacitive applications *etc.*, so present results (observed as the metallic-induced consequences of both phenomena) are significant attempt to open a route for tailoring the related properties by changing chemical modifiers instead of composition.

## Conflicts of interest

There are no conflicts to declare.

## Supplementary Material
